# Dose selection trial of metronomic oral vinorelbine monotherapy in patients with metastatic cancer: a hellenic cooperative oncology group clinical translational study

**DOI:** 10.1186/1471-2407-13-263

**Published:** 2013-05-29

**Authors:** Evangelos Briasoulis, Gerasimos Aravantinos, George Kouvatseas, Periklis Pappas, Eirini Biziota, Ioannis Sainis, Thomas Makatsoris, Ioannis Varthalitis, Ioannis Xanthakis, Antonios Vassias, George Klouvas, Ioannis Boukovinas, George Fountzilas, Kostantinos N Syrigos, Haralambos Kalofonos, Epaminontas Samantas

**Affiliations:** 1 Department of Medical Oncology, University of Ioannina, School of Medicine, Ioannina, Greece; 2 Second Department of Medical Oncology, “Agii Anargiri” Cancer Hospital, Athens, Greece; 3 Health Data Specialists Ltd, Athens, Greece; 4 Department of Pharmacology, University of Ioannina, Medical School, Ioannina, Greece; 5 University of Ioannina, Cancer Biobank Center, Ioannina, Greece; 6 Division of Oncology, Department of Medicine, University Hospital, University of Patras Medical School, Patras, Greece; 7 Oncology Department, General Hospital of Chania, Crete, Greece; 8 Department of Medical Oncology, “Papageorgiou” Hospital, Aristotle University of Thessaloniki School of Medicine, Thessaloniki, Greece; 9 Oncology Unit GPP, “Sotiria” General Hospital, Athens School of Medicine, Athens, Greece; 10 Second Department of Medical Oncology, “Metropolitan” Hospital, Piraeus, Greece; 11 Department of Medical Oncology, “Theagenio” Cancer Hospital, Thessaloniki, Greece; 12 Third Department of Medical Oncology, “Agii Anargiri” Cancer Hospital, Athens, Greece; 13 Current affiliation: Department of Hematology, School of Medicine, University of Ioannina, Ioannina, Greece

**Keywords:** Metronomic-chemotherapy, Antiangiogenic, Vinorelbine, Cancer

## Abstract

**Background:**

Metronomic chemotherapy is considered an anti-angiogenic therapy that involves chronic administration of low-dose chemotherapy at regular short intervals. We investigated the optimal metronomic dose of oral vinorelbine when given as monotherapy in patients with metastatic cancer.

**Methods:**

Patients with recurrent metastatic breast (BC), prostate (PC) or non-small cell lung cancer (NSCLC) and adequate organ functions were randomly assigned to 30, 40 or 50 mg vinorelbine, taken orally three times a week. Treatment continued until disease progression, unacceptable toxicity, withdrawal of consent or maximum 24 months. Primary endpoint was time-to-treatment failure (TTF) and secondary were progression-free survival (PFS), toxicity, changes in blood concentrations of angiogenesis-associated biomarkers and pharmacokinetics.

**Results:**

Seventy-three patients were enrolled. Four-month TTF rate did not differ between the three arms: 25.9% (11.1%-46.2% 95% Confidence Interval), 33.3% (15.6%-55.3%) and 18.2% (5.2%-40.3%) for the 30 mg, 40 mg and 50 mg arms (p-value = 0.56). Objective response was seen in 2 patients with NSCLC (treated at 30 and 50 mg respectively), one with BC (at 40 m g) and one with PC (at 50 mg) and lasted from 4 to 100 weeks, with maximum response duration achieved at 50 mg. Adverse events were mild and negligible and did not differ between the three arms. Blood levels of vinorelbine reached steady state from the second week of treatment and mean values for the 30, 40 and 50 mg were respectively 1.8 ng/ml (SD 1.10), 2.2 ng/ml (SD 1.87) and 2.6 ng/ml (SD 0.69). Low pre-treatment blood concentrations of FGF2 and IL8 predicted favorable response to therapy (p values 0.02 and 0.006, respectively), while high levels of *TEK* gene transcript predicted treatment resistance.

**Conclusions:**

Considering the antitumor activity and response duration, the negligible toxicity of the highest dose investigated and the lack of drug accumulation over time, we suggest that 50 mg given three times a week is the optimal dose for metronomic oral vinorelbine. Further investigation of metronomic oral vinorelbine (MOVIN) at this dose is warranted in combination with conventional chemotherapy regimens and targeted therapies.

**Trial registration:**

Clinicaltrials.gov NCT00278070

## Background

Systemic therapy of metastatic cancers has moderately progressed over the last decade. Conventional chemotherapy appears to have reached a plateau in efficacy for most major solid cancers [1,2] and a number of promising targeted therapeutics have failed to meet their objectives [3,4].

Metronomic chemotherapy (MC) has developed as a patient-friendly therapy on the concept to induce prolonged cancer control without significant side effects even in frail patients [5-9]. It involves chronic administration of low-dose chemotherapy at regular short intervals and mechanistically it stands between targeted anti-angiogenic therapy and conventional chemotherapy, complementing known shortcomings of both [10,11]. Most approved antiangiogenic drugs are inhibitors of the VEGF/VEGFR pathway of endothelial cells and demonstrate short-living clinical activity possibly because of rebound emergence of alternative angiogenic “escape” pathways [12-16]. Conventional application of cytotoxic drugs at maximum tolerated doses (MTD) aims to induce highest possible apoptosis on cancer cells, but it also affects healthy proliferating tissues and requests treatment-free intervals to allow recovery from toxicities. However these treatment gaps may facilitate repair of damaged tumor vasculature rendering cancers aggressive and resistant [17,18]. MC seems to have the capability to bridge weaknesses of both targeted antiangiogenic therapy and conventional chemotherapy because it induces a pathway-independent inhibition of function and proliferation of tumor endothelial cells and in addition it can damage cancer cells, restore immune response and induce tumor dormancy [11,19-21].

In a previously published phase 1A study we defined pharmacokinetics and a dose range of metronomic oral vinorelbine that can safely be given to patients with advanced cancer and provided clinical evidence of the antiangiogenic basis of this therapy [22]. We now report the results of a dose selection randomized trial registered at http://www.clinicaltrials.gov (Trial ID NCT00278070) that aimed to define the optimal metronomic dose of oral vinorelbine by considering clinical end points, pharmacokinetic data and correlative biomarkers. Defining the optimal dose of metronomic chemotherapy remains a challenge [23-26].

## Methods

### Study design

This was a multi-institutional randomized open-label phase IB trial conducted in 6 medical centers. Eligible patients were randomly assigned to receive oral vinorelbine tartrate (Navelbine® softgel capsules) at one of three predefined flat dose levels (30 or 40 or 50 mg) taken orally 3 times a week before lunch. Treatment continued until disease progression or occurrence of treatment related toxicity grade 3 or higher or patient’s decision or maximum 24 months treatment. The study was conducted in accordance with the Declaration of Helsinki and Scientific Committees of involved institutions approved the protocol.

### Patients

**Figure 1:**
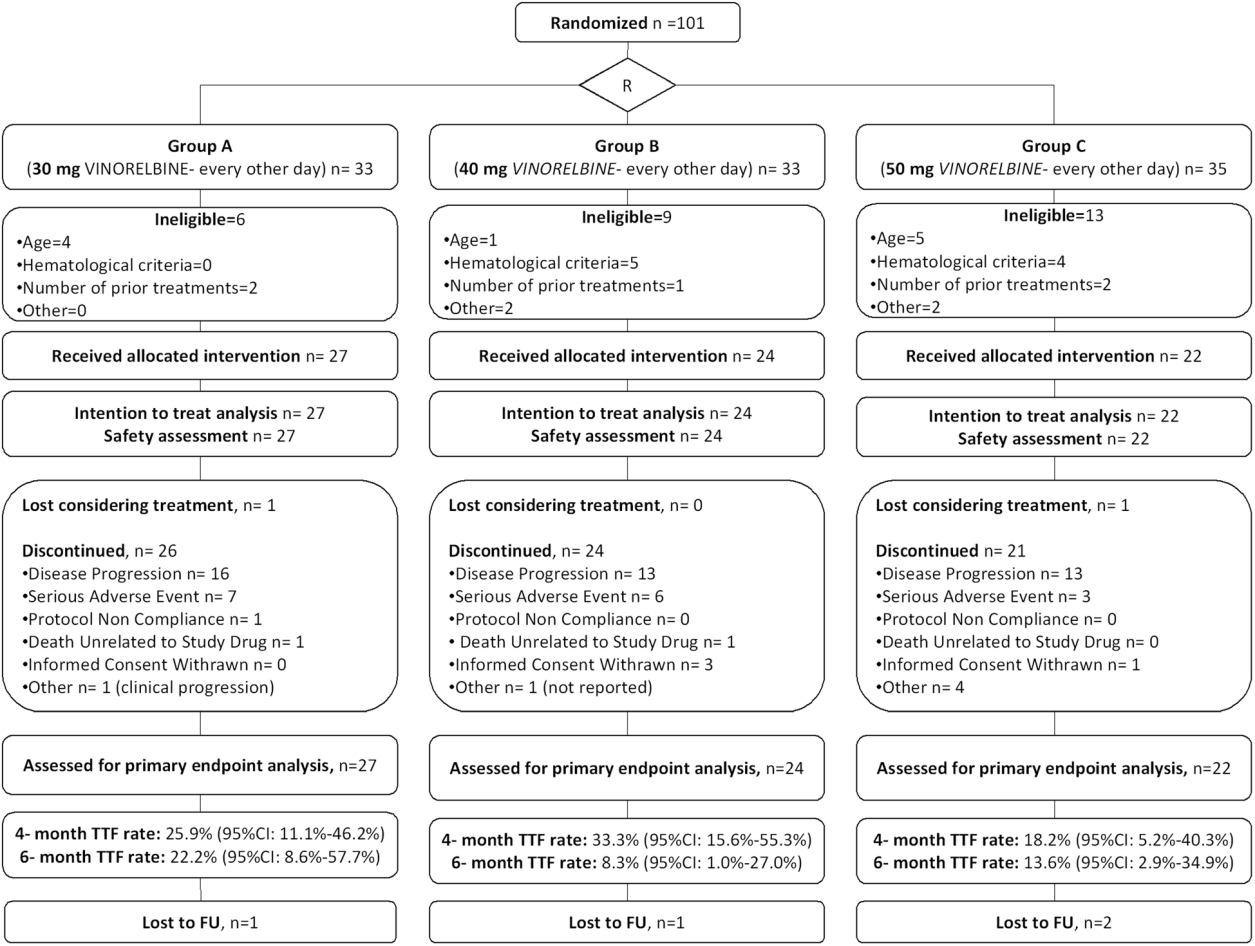
Consort diagram describing the main characteristics of the present clinical study.

Eligibility criteria were similar to those of the phase IA part of the trial [22]. Eligible patients had to sign an informed consent before participation. Eligibility criteria were as follows: age 16–75 years; performance status 0 to 2 according to the WHO scale; minimum life expectancy of 16 weeks; adequate bone marrow, hepatic and renal functions; absence of brain metastasis; metastatic and locally advanced hormonal refractory prostate, or previously treated metastatic breast cancer or non-small cell lung cancer previously treated with no more than two chemotherapeutic regimens; no other concurrent anticancer chemotherapy; serum creatinine within normal limits; hemoglobin of at least 10 g/L, white blood cell counts ≥3.5x10^9^/L; absolute neutrophil count (ANC) ≥1.5x10^9^/L; platelets ≥150x10^9/^L; total serum bilirubin ≤1.5× upper normal limit (UNL); transaminases ≤ 2.0 × upper normal limit (UNL) unless attributed to liver metastases. Concurrent limited field radiation therapy (RT) and any previous RT was allowed. Exclusion criteria were the following: major active infection; more than two prior chemotherapy regimens for metastatic disease; chemotherapy administered within 28 days prior to start of metronomic vinorelbine; extensive liver metastases occupying more than half the liver; ongoing anti-coagulation therapy; pregnancy or breastfeeding and any of the following if occurred within 12 months prior to randomization: myocardial infarction, severe/unstable angina, coronary/peripheral artery bypass graft, congestive heart failure, cerebrovascular accident or transient ischemic attack, pulmonary embolism, cardiac dysrhythmias of grade >/= 2, atrial fibrillation of any grade or heart rate corrected QT interval (QTc) > 450 msec for males or > 470 msec for females, uncontrolled hypertension (> 150/100 mmHg despite optimal medical therapy). Patients with severe acute or chronic medical or psychiatric condition that in the judgment of the investigator would make the patient inappropriate for entry into the trial would also be excluded. Drop-off reasons included serious adverse events possibly related to the study drug, conditions requiring therapeutic intervention not permitted by the protocol and patients personal preference. The consort diagram of the study is shown in Figure 1.

### Follow up and sampling

Patients attended outpatient clinics every two weeks during he first two months on treatment and monthly thereafter for clinical assessment and blood sampling. For pharmacokinetic analysis, 5 ml whole blood samples were collected into EDTA tubes (Sarstedt, Germany) before treatment initiation and at follow-up visits before taking oral vinorelbine. Samples were stored at −20°C until analysis. Additional blood plasma and blood RNA were obtained prior to treatment, 4 weeks later and on a monthly schedule in consented patients in order to study circulating angiogenesis biomarkers. Both plasma and PAXgene RNA™ tubes (Qiagen, Thessaloniki, Greece) were stored at −80°C until analysis.

### Study endpoints

The primary clinical endpoint was time to treatment failure (TTF). TTF rates per treatment arm would be compared at 4 and 6 months. Secondary endpoints were progression-free survival (PFS), time-to-progression, toxicity, correlation of baseline blood concentrations of angiogenesis-associated surrogate markers with treatment efficacy measures, and pharmacokinetics.

Toxicity was evaluated according to the National Cancer Institute Common Toxicity Criteria V3. Acute toxicity was considered any adverse event that occurred during the first 8 weeks of treatment, while chronic toxicity was characterized any side effect that was recorded four months after the initiation of treatment. Toxicity that occurred between 8 weeks to 4 months of treatment was characterized sub-acute.

Treatment response was evaluated in patients that had completed at least 6 weeks of treatment and had at least one follow-up tumor assessment. Baseline tumor assessment was performed within 4 weeks prior to treatment initiation and thereafter every 2 months until documentation of response. Documented response should be confirmed after 4 weeks and should be regularly assessed every 4 months thereafter. Chest X-rays, computerized tomographic (CT) scans, ultrasound imaging studies and clinical measurements were used as appropriate. Response was documented using the RECIST response criteria for solid tumors [27] and Bubley Criteria for prostate cancer [28].

### Circulating biomarkers

**Figure 2:**
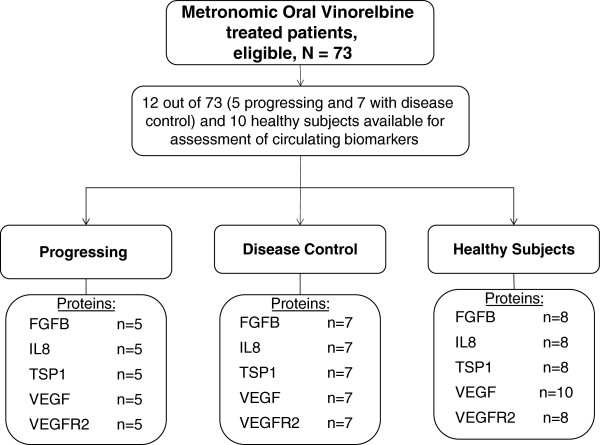
REMARK diagram for biomarker studies.

Plasma concentrations of basic fibroblast growth factor (FGF2), vascular endothelial growth factor-A (VEGFA), interleukin-8 (IL8) and thrombospondin-1 (TSP1) were determined by using commercially available quantitative sandwich enzyme immunoassays. In particular, Quantikine kits (R&D Systems, Inc., Minneapolis, USA) were used for FGF2, VEGF, VEGFR2 and IL8, and the ChemiKine Human TSP1 EIA Kit for TSP1 (Chemicon International, Inc., Temecula, CA, USA). Protocols, procedures, and equipment were used according to the manufacturer’s instructions. The lower detection limits for FGF2, VEGF, VEGFR2, IL8 and TSP1 were respectively 10, 31.2, 78.1, 31.2 and 9.8 pg/mL and the means for intra- and inter-assay coefficients of variation were 3.6 to 7.8 and 6.5 to 10.0, respectively. Optical densities were determined using a microfilter plate reader (DAS-A3, Roma, Italy) with filters for 450 nm (IL8, VEGF, VEGFR-2) and 490 nm (FGF2, TSP1). All analyses were carried out in duplicate. Samples available for each biomarker are shown in the REMARK diagram of Figure 2[29].

### RNA extraction, reverse transcription and real time PCR arrays

Peripheral blood was drawn in PAXgene tubes and kept frozen at −80°C. Total RNA was extracted with the PAXgene blood RNA kit, according to the manufacturer’s instructions (PreAnalytiX/Qiagen), within 6 months from collection. The additive contained in the PAXgene tubes reduces *in vitro* RNA degradation and minimizes *in vitro* gene induction [30]. RNA was extracted from 4 patients who either had an objective response (three patients) or TTF >6 months (1 patient) and 4 patients who relapsed early (within 2 months of the initiation of treatment). The analysis was done in mirrored samples: before treatment initiation and 4 weeks after treatment initiation. RNA integrity was checked by both conventional RNA electrophoresis and Agilent bioanalizer 2100. High quality RNA was reverse transcribed and analyzed on a PCR array platform (PAHS-024 F, SABiosciences) and the expression levels of 84 genes involved in angiogenesis were determined by using real time PCR.

Only RNAs with RNA integrity number (RIN) >7 were used for reverse transcription and further processing. For reverse transcription the SABiosciences RT^2^ First Strand kit was used. In all cases RNA of 0.5 μg was reversed transcribed. Simultaneous quantification of 84 key genes involved in angiogenesis was done by using the angiogenesis RT^2^ profiler PCR Array (PAHS-024 F, SABiosciences). Relative expression was determined with the Light Cycler 480 instrument (Roche) and the ΔΔCt method [31].

### Pharmacokinetics

Whole blood samples were shipped on dry ice to the Institute de Recherché Pierre Fabre, Castres, France, for analysis. Concentrations of vinorelbine (VRL) and its main metabolite, 4-O-deacetylvinorelbine (DVRL) were quantified using a sensitive LC/MS/MS method previously reported [32].

### Statistical analysis

Analysis was performed on an intent-to-treat basis. Regarding the definition of endpoints reported, time to treatment failure (TTF) was calculated as the time from random assignment to disease progression or death from any cause or early treatment discontinuation. Time to progression (TTP) was calculated as the time from random assignment to disease progression. Progression-free survival (PFS) was calculated as the time from random assignment to disease progression or death from any cause. Survival was calculated as the time from random assignment to death from any cause. Event-free patients at last contact were censored. Time-to-event distributions were estimated using Kaplan-Meier curves while the log-rank test was used for comparisons.

The Fisher’s exact test and the Jonckheere-Terpstra exact test were used to examine differences in toxicity rates between treatment groups. Association between biomarkers at baseline and response/activity (patients without objective response and/or progressing within 4 months vs those with objective response or PFS > 4 months vs healthy controls) was assessed with the Kruskall-Wallis test. Correlations among biomarkers were calculated using the Pearson’s correlation test. Significance was determined at the level of 5% (two-sided).

The sample size for this 3-arm randomized study was estimated under the assumption that the expected 4 and 6 month TTF rate for the 30 mg, 40 mg and 50 mg groups would be 10%, 30% and 50% and 5%, 20% and 40% respectively. Using a global chi-square test at the 2.5% level of significance (Bonferroni adjusted) the study had 80% power to reject the null hypothesis that all the arms had the same TTF rate [33].

## Results

Between January 2006 and January 2007, 73 eligible patients were recruited into the trial. The median age of the patients was 67 years for the 30 and 50 mg dose levels and 66 years for the 40 mg dose level arm. The majority of patients (68.5%) were male, they had a PS 1–2 (64.4%) and the most common cancer was NSCLC (42.5%), while 80.8% had received prior chemotherapy and the most common metastatic site were bones (57.5%). Baseline patients’ characteristics are shown in Table 1.

**Table 1:** Patients’ characteristics

		**Oral vinorelbine dose arms**		
		**30 mg**	**40 mg**	**50 mg**
**Patients**	*N*	27	24	22
**Age**	*median*	67	66	67
	*range*	43-74	48-75	44-75
**Gender**	*Male*	18 (66.6%)	17 (70.8%)	15 (68.2%)
	*Female*	9 (33.4%)	7 (29.2%)	7 (31.8)
**Cancer type**	*Breast*	7 (26.0%)	6 (25.0%)	7 (31.8%)
	*Prostate*	8 (29.6%)	7 (29.2%)	7 (31.8%)
	*NSCLC*	12 (44.4%)	11 (45.8%)	8 (36.4%)
**PS**	*0*	8 (29.6%)	10 (41.6%)	8 (36.4%)
	*1*	15 (55.6%)	11 (45.8%)	12 (54.6%)
	*2*	4 (14.8%)	3 (12.6%)	2 (9.0%)
**Prior chemotherapy**	*0*	5 (18.6%)	4 (16.6%)	5 (22.8%)
	*1*	13 (48.2%)	12 (50.0%)	11 (50.0%)
	*2*	9 (33.4%)	8 (33.4%)	6 (27.2%)
**Prior radiotherapy**		14 (51.8%)	8 (33.4%)	11 (50.0%)
**Prior hormonotherapy**		12 (44.4%)	11 (44.8%)	13 (59.0%)
**Organs with metastases**	*lung*	15 (55.6%)	14 (58.4%)	10 (45.4%)
	*liver*	8 (29.6%)	4 (16.6%)	3 (13.6%)
	*bone*	18 (66.6%)	15 (62.6%)	9 (41%)

### Clinical outcomes

**Figure 3:**
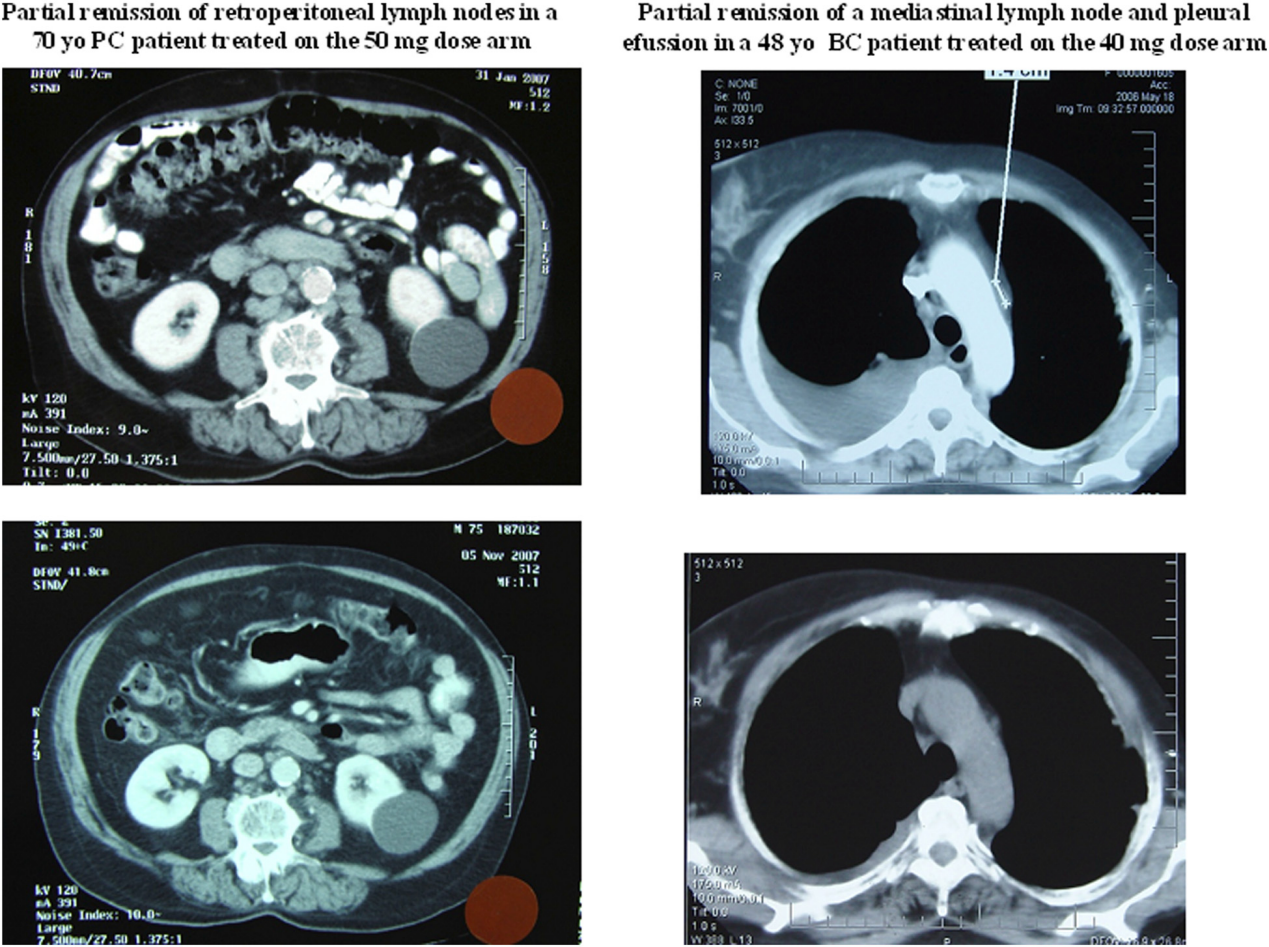
**Objective tumor responses.** Objective responses documented in a prostate cancer patient treated at 50 mg dose and a breast cancer patient treated at 40 mg dose.

**Figure 4:**
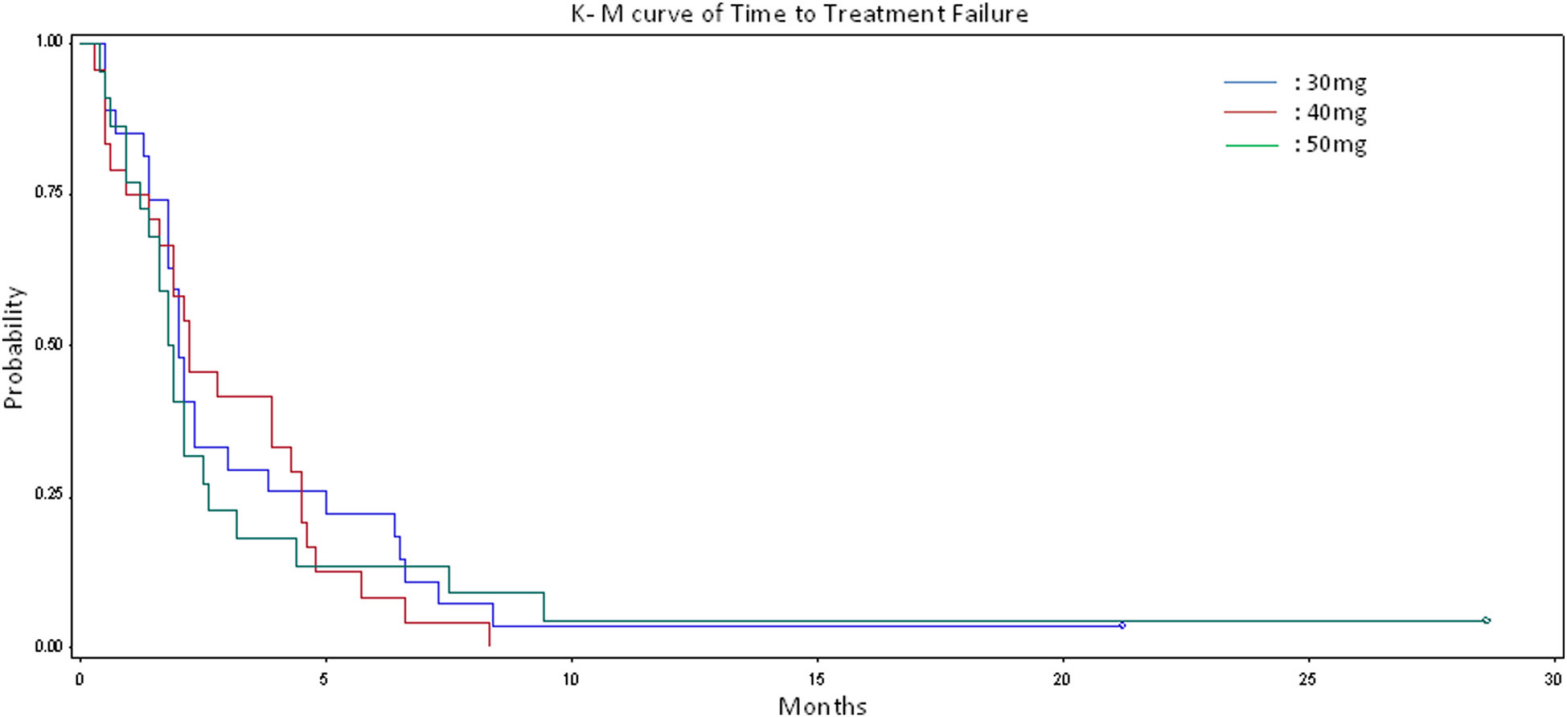
Time to treatment failure probability curve.

Antitumor efficacy was seen at all dose arms. Confirmed partial remissions were documented in four cases of patients. Those were two NSCLC patients who received 30 mg (response duration 4 weeks) and 50 mg (response duration 100 weeks) one breast cancer patient treated at 40 mg (response duration 18 weeks) and one prostate cancer patient treated at 50 mg (response duration 30 weeks) (Figure 3). Eleven patients achieved a TTF longer than 6 months. No arm was found superior at the primary endpoint (Table 2, Figure 4). The median time to treatment failure was eight weeks in all three arms, while the 4 month TTF rate point estimates and 95% Confidence Intervals (CIs) for the 30 mg, 40 mg and 50 mg vinorelbine groups were 25.9% (11.1%-46.2%), 33.3% (15.6%-55.3%) and 18.2% (5.2%-40.3%) respectively. The p-value of the Fisher’s exact test for equality of the rates is 0.56. Similarly, the 6 month TTF rate point estimates and 95% CIs were respectively 22.2% (8.6%-57.7%), 8.3% (1.0%-27.0%) and 13.6% (2.91%-34.9%) for the 30 mg, 40 mg and 50 mg groups respectively. The *p*-value of the Fisher’s exact test is 0.39. In both cases the TTF rate did not vary statistically significant among the treatment arms. No differences were noticed among the three arms with regard to PFS and overall survival.

**Table 2:** TTF rate point estimates and 95% confidence intervals for the 30 mg, 40 mg and 50 mg vinorelbine groups (TTF refers to time from treatment initiation to discontinuation for any reason)

	**Patients (%) on treatment**			
**Vinorelbine dose arm (mg)**	**At 4 months**		**At 6 months**	
		***p value***		***p value***
30	25.9% (11.1-46.2%)	0.56	22.2% (8.6-57.7%)	0.39
40	33.3% (15.6-55.6%)		8.3% (1.0-27.0%)	
50	18.2% (5.2-20.3%)		13.6% (2.9-34.9%)	

The side effects observed were generally mild and negligible. No differences in grade 3 and 4 acute toxicities were seen among the three dose levels. The only statistically significant difference observed was lymphopenia (worse in lower dose levels) and gastrointestinal (worse at dose level 40) (Table 3). Chronic toxicities did not occur.

**Table 3:** Worse toxicity events by grade and treatment dose group- N of patients

**Label of variable**	**Treatment dose group**												***FISH test p value***
	**Vinorelbine 30mg**				**Vinorelbine 40mg**				**Vinorelbine 50mg**				
	**Grade**				**Grade**				**Grade**				
	**1**	**2**	**3**	**4**	**1**	**2**	**3**	**4**	**1**	**2**	**3**	**4**	
**Hemoglobin**	17	2	1	.	16	3	1	.	14	2	.	.	ND
**Leukocytes**	5	.	1	2	2	3	1	2	3	3	2	1	ND
**Neutrophils**	1	1	2	1	5	1	1	2	3	1	2	2	ND
**Platelets**	4	.	.	.	1	.	.	.	1	.	.	.	ND
**Neuropathy Sensor**	1	1	.	.	2	.	.	.	3	.	.	.	ND
**Diarrhea**	2	.	.	.	1	.	1	.	.	.	.	.	ND
**Constipation**	2	.	.	.	2	.	.	.	1	.	1	.	ND
**Fatigue**	7	.	.	.	5	.	1	.	4	.	.	1	ND
**Metabolic/Laboratory**	6	1	1	.	3	2	1	.	5	1	.	.	ND
**Pain**	1	2	2	.	6	.	.	.	2	1	.	.	ND
**Hemoptysis**	.	1	.	.	1	.	.	.	.	.	.	.	ND
**Edema: limb**	.	.	.	.	1	.	1	.	1	.	1	.	ND
**Upper Respiratory**	3	.	2	.	3	.	.	.	.	.	.	.	ND
**Febrile Neutropenia**	.	.	.	.	.	.	1	.	.	.	.	.	ND
**Gastrointestinal**	1	.	.	.	6	2	.	.		1	.	.	0,035
**Weight (Gain- Loss)**	.	2	1	.	1	.	.	.	.	.	.	.	ND
**Fever**	.	1	.	.	1	.	.	.	1	1	.	.	ND
**Lymphopenia**	.	3	.	.	2	1	1	.	.	.	.	.	0,032

### Pharmacokinetics

**Figure 5:**
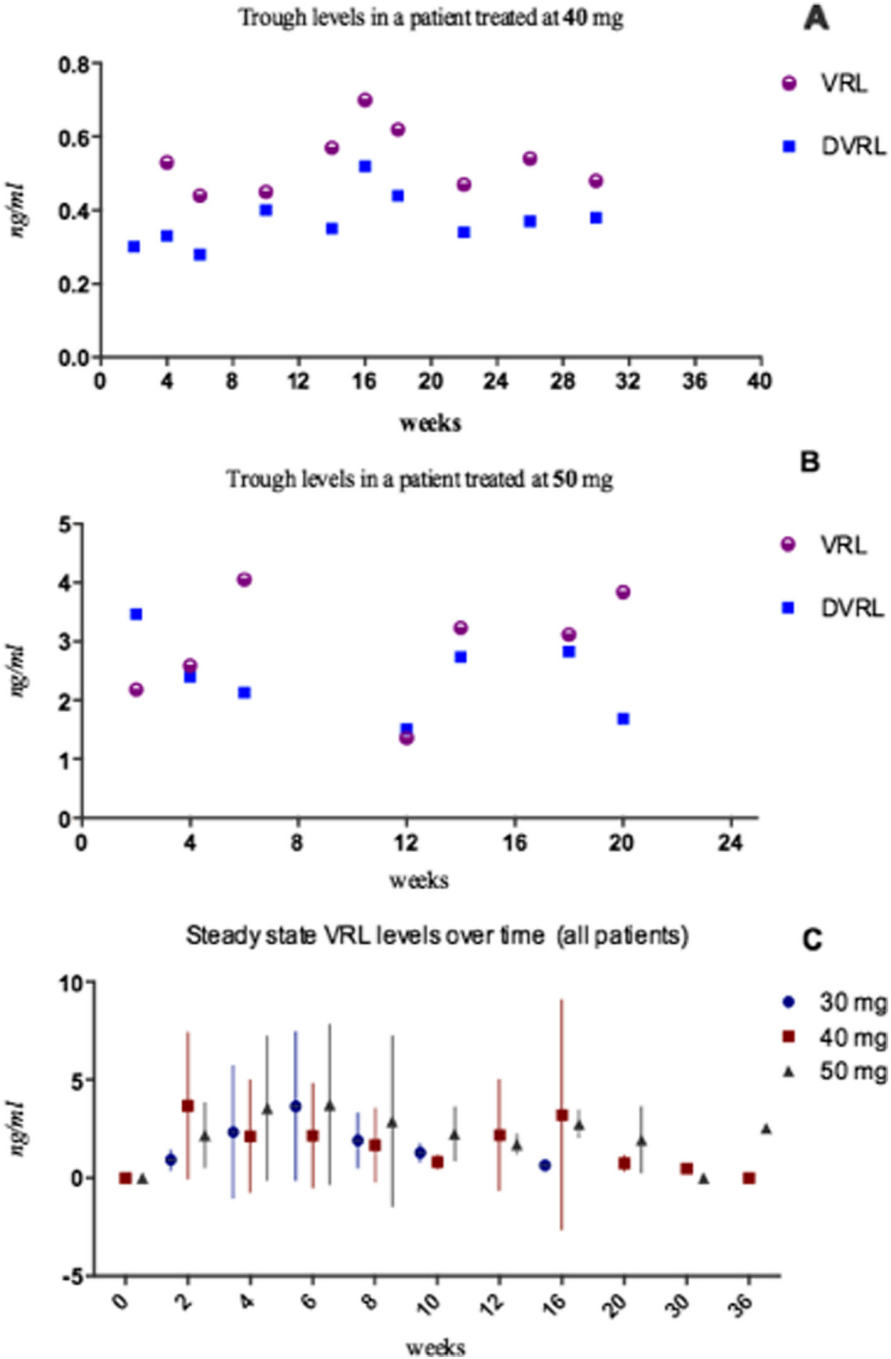
**Trough blood concentrations of vinorelbine over time.** Scatter plots of serial trough concentrations of VLR and its active metabolite DVLR over time in a patient prostate cancer patient treated at 50 mg dose arm (**A**) and a breast cancer patient treated at 40 mg (**B**) and median (plus SEM) values of steady state levels of all patients treated at the three dose arms (**C**).

Trough levels of vinorelbine were measured in 237 blood samples drawn from 44 consented patients (61% of treated patients) over a time that spanned duration of therapy from 2 to 36 weeks. Steady state concentrations were similar to those recorded in the phase IA trial with no evidence of accumulation over time (Figure 5). Mean values and standard deviation (SD) for the 30 mg dose was 1.8 ng/ml (SD 1.10), 2.2 ng/ml (SD 1.87) for the 40 mg dose and 2.6 ng/ml (SD 0.69) for the 50 mg dose. A dose proportional increase of steady state concentrations was noted but did not reach statistical significance (ANOVA p value = 0.5).

### Circulating angiogenesis modulating proteins and gene transcripts

Of the investigated angiogenesis modulating proteins, baseline pre-treatment levels of FGF2 and IL8 were found to have a power to predict favorable response to metronomic vinorelbine (p value = 0.02 and 0.006 respectively). In particular, patients with baseline levels approximating those of healthy individuals had a chance to have a favorable outcome to therapy. VEGF and VEGFR2 baseline levels did not differ between patients with favorable versus non-favorable therapy outcome and TSP-1 data were inconclusive at a marginal p-value (Table 4).

**Table 4:** Assessment of circulating angiogenesis modulating proteins as therapy activity predictors

		**Treatment failure**	**Objective response or TTF > 4monts**	**Healthy controls**
**FGF2 (pg/ml)**	*N*	5	7	8
	*median*	3.4	1.8	0.8
	*range*	(1.9-23.7)	(0.4-14.8)	(0.2-2.3)
	***p-value***			0.02*
**IL8 (pg/ml)**	*N*	5	7	8
	*median*	27.2	5.8	5.8
	*range*	(12.2-61.9)	(2.8-18.9)	(1.1-7.5)
	***p-value***			0.006**
**TSP-1 (ng/ml)**	*N*	5	7	8
	*median*	125.1	69.6	67.3
	*range*	(85.1-328.4)	(24-452)	(9-710.1)
	***p-value***			***0.047***
**VEGF (pg/ml)**	*N*	5	7	10
	*median*	1552	428,6	2094
	*range*	(804.3-2785)	(252.4-3116)	(1122-2387)
	*p-value*			*0.074*
**VEGFR-2 (pg/ml)**	*N*	5	7	8
	*median*	13680	13275	12328
	*range*	(8945-16465)	(7455-18065)	(8235-17615)
	*p-value*			*0.657*

**Figure 6:**
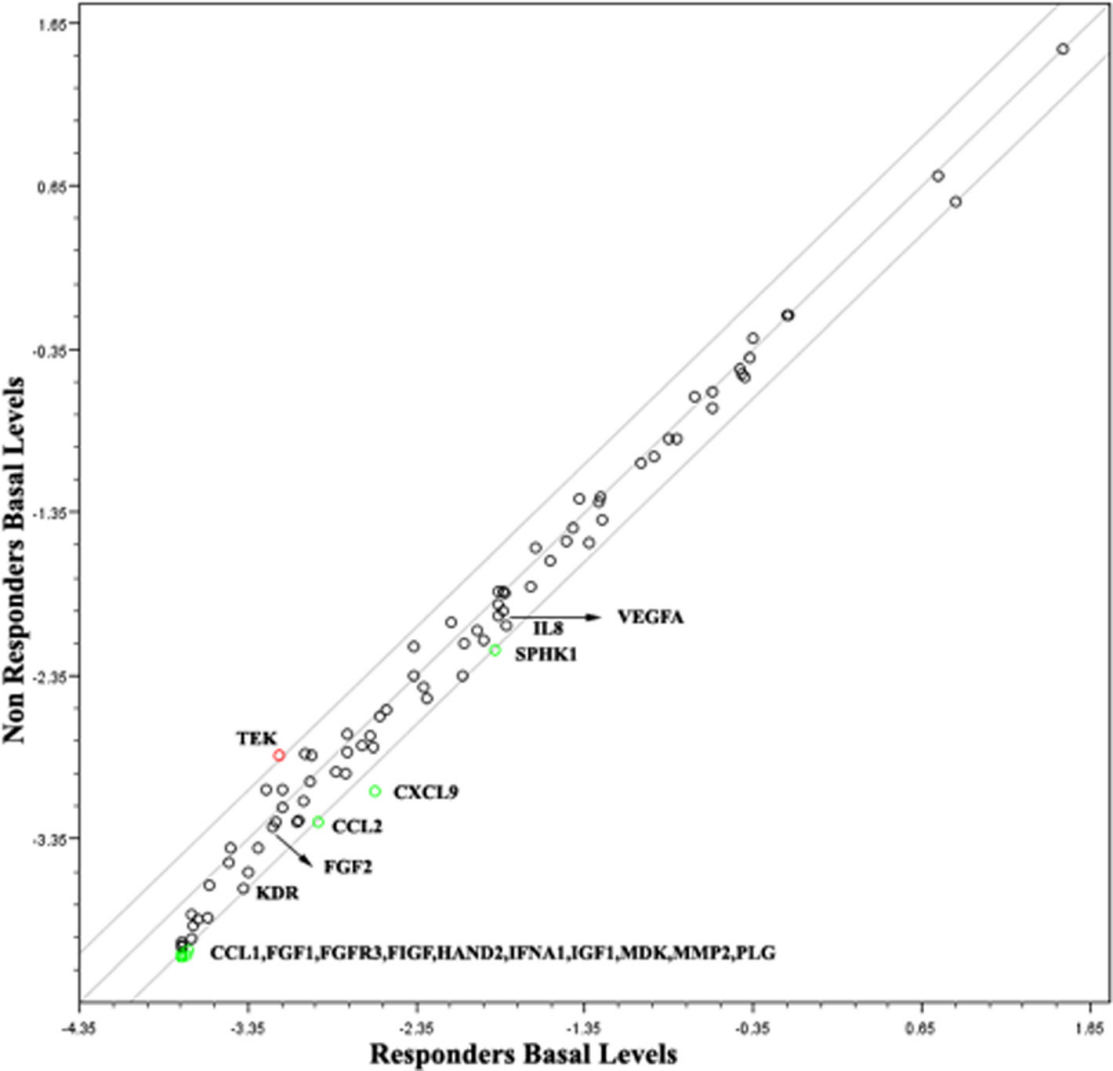
**Transcript levels of 84 angiogenesis-related genes graphed against treatment outcome Scatter plots comparing circulating pretreatment gene transcript levels of responders versus non-responders.** The graphs plot the log_10_ of normalized gene expression levels between the two conditions, responders (x-axis) and non-responders (y-axis). Dots outside the area between the two lines indicate fold differences larger than a threshold of 2. The red dots in the upper left corner readily identify up-regulated genes, and the green dots in the lower right corner readily identify down regulated genes.

Data analysis of circulating gene transcripts by using the ΔΔCt method revealed that TEK gene transcript was significantly up-regulated before treatment in non responders, while gene transcripts of SPHK1, CCL2, KDR, CCL1, FGF1, FGFR3, FIGF, HAND2, IFNA1, IGF1, MDK, MMP2, PLG were down-regulated about two fold and the CXCL9 gene was down-regulated 2.92 fold. FGF2 transcript post treatment initiation was found to be up-regulated in non responders and IL8, PGF, FIGF, IGF1 gene transcripts were down-regulated more than 2 folds (Figure 6).

## Discussion

This was a dose selection study of metronomic oral vinorelbine for the treatment of metastatic NSCLC, breast and prostate cancer patients that had relapsed from a previous therapy. The primary clinical endpoint of this trial was set to be time to treatment failure (TTF). We chose TTF as a primary endpoint because it is a composite measure that co-estimates time from randomization to discontinuation of treatment for any reason, including disease progression, treatment toxicity, and death. It trade-offs efficacy and toxicity, which is of major importance for metronomic therapy, especially when tested in patients with diverse tumor types [34]. This trial failed to demonstrate superiority in any of the three doses investigated at the primary endpoint, since median TTF was six weeks for all three arms and the TTF rates at 4 and 6 months did not differ among the three arms either. Considering the objective tumor responses, partial remission was confirmed in four cases and lasted up to 49 weeks. Two of the objective responses occurred in patients treated at the upper dose arm and lasted longer compared to objective responses that were achieved with the lower doses. Major finding of this trial, as in the previous one, was that activity came at no cost of clinically significant toxicity, which is a hallmark of metronomic chemotherapy [19]. In addition, toxicities that are known to occur with drugs that inhibit the VEGF – VEGFR-2 pathway were not seen with metronomic vinorelbine, which supports that a combination of these two antiangiogenic therapies would be probably feasible [35,36].

Antiangiogenic therapy is known to work optimally if endothelial cells are exposed to steady levels of inhibitors [11,37]. Both vinorelbine and its active metabolite achieved steady state concentrations at the low nanomolar range, which was found in vitro to optimally inhibit proliferation of endothelial cell and induce expression of endogenous anti-angiogenic molecules [38].

Similarly to the phase IA study, we found that low baseline levels of circulating endogenous promoters of angiogenesis and in particular IL8 and FGF2 could predict clinical benefit from treatment with metronomic vinorelbine [22]. In addition, we investigated circulating transcripts of a panel of 84 key genes involved in modulating the biological processes of angiogenesis. Among them, high baseline levels of TEK transcripts were associated with resistance to therapy. TEK, a biomareker of hemangioblasts, appears to be a potential predictor of refractoriness to antiangiogenic therapy [39,40]. This is possibly related to its function as an endothelial cell surface receptor for angiopoietins ANGPT1, ANGPT2 and ANGPT4, that are regulators of survival and adhesion of endothelial cells and promoters of vascular stability and quiescence [41].

Overall, this study failed to specify the optimal metronomic dosage of oral vinorelbine by the primary endpoint. However it boosts the findings of the phase IA dose-ranging study by confirming that metronomic oral vinorelbine can safely be administered at doses up to 50 mg three time a week and that it can yield long lasting antitumor activity at the this dose without overt toxicity. In addition the steady-state nanomolar concentrations and the association of angiogenesis modulating factors with its activity denotes that the mechanism of antitumor activity of metronomic therapy is most likely antiangiogenic, as suggested by other investigators too [26,42].

## Conclusion

In conclusion, by taking into consideration the antitumor activity and response duration, negligible toxicity of the higher dose tested and lack of drug accumulation over time, we suggest that metronomic oral vinorelbine warrants further investigation in combination with conventional chemotherapy regimens or targeted angiogenic therapies at the dose of 50 mg given every other day, three times a week. Finally these data add support to the concept that metronomic scheduling provides a new future for using cytotoxic chemotherapy, especially in cases of incurable metastatic cancers [43].

## Abbreviations

ANC: Absolute neutrophil count; ANOVA: analysis of variance; ANGPT1: Angiopoietin 1; ANGPT2: Angiopoietin 2; ANGPT4: Angiopoietin 4; CCL1: Chemokine ligand 1; CCL2: Chemokine ligand 2; CXCL9: Chemokine (C-X-C motif) ligand 9; BC: Breast; CI: Confidence interval; CTC: Common toxicity criteria; CT: Computerized tomography; EDTA: Ethylenediaminetetraacetic acid; FGF1: fibroblast growth factor 1 (acidic); FGF2: fibroblast growth factor 2 (basic); FIGF: C-fos induced growth factor; HAND2: Heart and neural crest derivatives expressed 2; IGF1: Insulin-like growth factor 1; IL8: Interleukin-8; IFNA1: Interferon alpha-1; LC/MS/MS: Liquid chromatography - tandem mass spectrometry; MDK: Midkine (neurite growth-promoting factor 2); MC: Metronomic chemotherapy; MMP2: Matrix metalloproteinase-2; NSCLC: Non-small cell lung cancer; MTD: Maximum tolerated doses; PC: Prostate cancer; PLG: Plasminogen; PGF: Placental growth factor; PS: Performance status; QTc: QT interval corrected; RNA: Ribonucleic acid; TSP1: Thrombospondin-1; TTF: Time-to-treatment failure; PFS: Progression-free survival; PCR: Polymerase Chain Reaction; RECIST: Response Evaluation Criteria in Solid Tumors; RIN: RNA integrity number; RT: Radiation therapy; SD: Standard deviation; SPHK1: Sphingosine kinase 1; TEK: Tyrosine kinase, endothelial; VRL: Vinorelbine; DVRL: 4-O-deacetylvinorelbine; VEGFA: Vascular endothelial growth factor-A; VEGFR-2: Vascular endothelial growth factor receptor 2; WHO: world health organization.

## Competing interest

Evangelos Briasoulis received a research fund from Pierre Fabre, Farmaka SA, Greece, through the Research Committee of the University of Ioannina, for the biomarkers subproject of the study. Other authors declare no conflicts of interest.

## Authors’ contributions

Conception: EB. Study Design: EB, GK, PP. Patient enrollment, clinical management, data recording and collection of biologic material: EB, GA, TM, IV, IX, AV, GK, IB, GF, KNS, HK, ES. Molecular analysis and interpretation: IS. Pharmacokinetics and biomarkers analysis and interpretation: PP, EB. Statistical design and analysis: GK. All authors read and approved the final manuscript.

## Pre-publication history

The pre-publication history for this paper can be accessed here:

http://www.biomedcentral.com/1471-2407/13/263/prepub

## References

[B1] Kosmidis PA Fountzilas G Eleftheraki AG Kalofonos HP Pentheroudakis G Skarlos D Dimopoulos MA Bafaloukos D Pectasides D Samantas E Paclitaxel and gemcitabine versus paclitaxel and vinorelbine in patients with advanced non-small-cell lung cancer. A phase III study of the hellenic cooperative oncology group (HeCOG) Ann Oncol 2011 22 4 827 834 10.1093/annonc/mdq445 20880999

[B2] Saltz LB Progress in cancer care: the hope, the hype, and the gap between reality and perception J Clin Oncol 2008 26 31 5020 5021 10.1200/JCO.2008.17.6198 18794538

[B3] Fojo T Parkinson DR Biologically targeted cancer therapy and marginal benefits: are we making too much of too little or are we achieving too little by giving too much? Clin Cancer Res 2010 16 24 5972 5980 10.1158/1078-0432.CCR-10-1277 21169250

[B4] Richards L Targeted therapies: disappointing outcomes for anti-VEGF therapy Nat Rev Clin Oncol 2011 8 4 194 21451496 10.1038/nrclinonc.2011.28

[B5] Gebbia V Serretta V Borsellino N Valerio MR Salvage therapy with oral metronomic cyclophosphamide and methotrexate for castration-refractory metastatic adenocarcinoma of the prostate resistant to docetaxel Urology 2011 78 5 1125 1130 10.1016/j.urology.2011.08.010 22054386

[B6] Kerbel RS Klement G Pritchard KI Kamen B Continuous low-dose anti-angiogenic/ metronomic chemotherapy: from the research laboratory into the oncology clinic Ann Oncol 2002 13 1 12 15 10.1093/annonc/mdf093 11863092

[B7] Mir O Domont J Cioffi A Bonvalot S Boulet B Le Pechoux C Terrier P Spielmann M Le Cesne A Feasibility of metronomic oral cyclophosphamide plus prednisolone in elderly patients with inoperable or metastatic soft tissue sarcoma Eur J Cancer 2011 47 4 515 519 10.1016/j.ejca.2010.11.025 21251814

[B8] Penel N Clisant S Dansin E Desauw C Degardin M Mortier L Vanhuyse M Bonodeau F Fournier C Cazin JL Megestrol acetate versus metronomic cyclophosphamide in patients having exhausted all effective therapies under standard care Br J Cancer 2010 102 8 1207 1212 10.1038/sj.bjc.6605623 20354522 PMC2856003

[B9] Wong NS Buckman RA Clemons M Verma S Dent S Trudeau ME Roche K Ebos J Kerbel R Deboer GE Phase I/II trial of metronomic chemotherapy with daily dalteparin and cyclophosphamide, twice-weekly methotrexate, and daily prednisone as therapy for metastatic breast cancer using vascular endothelial growth factor and soluble vascular endothelial growth factor receptor levels as markers of response J Clin Oncol 2010 28 5 723 730 10.1200/JCO.2009.24.0143 20026801

[B10] Hahnfeldt P Folkman J Hlatky L Minimizing long-term tumor burden: the logic for metronomic chemotherapeutic dosing and its antiangiogenic basis J Theor Biol 2003 220 4 545 554 10.1006/jtbi.2003.3162 12623285

[B11] Kerbel RS Kamen BA The anti-angiogenic basis of metronomic chemotherapy Nat Rev Cancer 2004 4 423 436 10.1038/nrc1369 15170445

[B12] Yancopoulos GD Clinical application of therapies targeting VEGF Cell 2010 143 1 13 16 10.1016/j.cell.2010.09.028 20887885

[B13] Tejpar S Prenen H Mazzone M Overcoming resistance to antiangiogenic therapies Oncologist 2012 17 8 1039 1050 10.1634/theoncologist.2012-0068 22773560 PMC3425522

[B14] Huang D Ding Y Zhou M Rini BI Petillo D Qian CN Kahnoski R Futreal PA Furge KA Teh BT Interleukin-8 mediates resistance to antiangiogenic agent sunitinib in renal cell carcinoma Cancer Res 2010 70 3 1063 1071 10.1158/0008-5472.CAN-09-3965 20103651 PMC3719378

[B15] Ellis LM Hicklin DJ VEGF-targeted therapy: mechanisms of anti-tumour activity Nat Rev Cancer 2008 8 8 579 591 10.1038/nrc2403 18596824

[B16] Bergers G Hanahan D Modes of resistance to anti-angiogenic therapy Nat Rev Cancer 2008 8 8 592 603 10.1038/nrc2442 18650835 PMC2874834

[B17] Shaked Y Kerbel RS Antiangiogenic strategies on defense: on the possibility of blocking rebounds by the tumor vasculature after chemotherapy Cancer Res 2007 67 15 7055 7058 10.1158/0008-5472.CAN-07-0905 17671170

[B18] Frei E 3rd Canellos GP Dose: a critical factor in cancer chemotherapy Am J Med 1980 69 4 585 594 10.1016/0002-9343(80)90472-6 6999898

[B19] Pasquier E Kavallaris M Andre N Metronomic chemotherapy: new rationale for new directions Nat Rev Clin Oncol 2010 7 8 455 465 10.1038/nrclinonc.2010.82 20531380

[B20] Chen CA Ho CM Chang MC Sun WZ Chen YL Chiang YC Syu MH Hsieh CY Cheng WF Metronomic chemotherapy enhances antitumor effects of cancer vaccine by depleting regulatory T lymphocytes and inhibiting tumor angiogenesis Mol Ther 2010 18 6 1233 1243 10.1038/mt.2010.34 20372107 PMC2889744

[B21] Andre N Padovani L Pasquier E Metronomic scheduling of anticancer treatment: the next generation of multitarget therapy? Future Oncol 2011 7 3 385 394 10.2217/fon.11.11 21417902

[B22] Briasoulis E Pappas P Puozzo C Tolis C Fountzilas G Dafni U Marselos M Pavlidis N Dose-ranging study of metronomic oral vinorelbine in patients with advanced refractory cancer Clin Cancer Res 2009 15 20 6454 6461 10.1158/1078-0432.CCR-09-0970 19808873

[B23] Skipper HE Schabel FM Jr Mellett LB Montgomery JA Wilkoff LJ Lloyd HH Brockman RW Implications of biochemical, cytokinetic, pharmacologic, and toxicologic relationships in the design of optimal therapeutic schedules Cancer Chemother Rep 1970 54 6 431 450 5527023

[B24] DeVita VT Jr Chu E A history of cancer chemotherapy Cancer Res 2008 68 21 8643 8653 10.1158/0008-5472.CAN-07-6611 18974103

[B25] Maraveyas A Lam T Hetherington JW Greenman J Can a rational design for metronomic chemotherapy dosing be devised? Br J Cancer 2005 92 8 1588 1590 10.1038/sj.bjc.6602474 15846302 PMC2362004

[B26] Shaked Y Emmenegger U Man S Cervi D Bertolini F Ben-David Y Kerbel RS Optimal biologic dose of metronomic chemotherapy regimens is associated with maximum antiangiogenic activity Blood 2005 106 9 3058 3061 10.1182/blood-2005-04-1422 15998832 PMC1895327

[B27] Therasse P Arbuck SG Eisenhauer EA Wanders J Kaplan RS Rubinstein L Verweij J Van Glabbeke M van Oosterom AT Christian MC New guidelines to evaluate the response to treatment in solid tumors. European organization for research and treatment of cancer, national cancer institute of the United States, national cancer institute of Canada J Natl Cancer Inst 2000 92 3 205 216 10.1093/jnci/92.3.205 10655437

[B28] Bubley GJ Carducci M Dahut W Dawson N Daliani D Eisenberger M Figg WD Freidlin B Halabi S Hudes G Eligibility and response guidelines for phase II clinical trials in androgen-independent prostate cancer: recommendations from the prostate-specific antigen working group Journal of clinical oncology : official journal of the American Society of Clinical Oncology 1999 17 11 3461 3467 10550143 10.1200/JCO.1999.17.11.3461

[B29] McShane LM Altman DG Sauerbrei W Taube SE Gion M Clark GM Reporting recommendations for tumor marker prognostic studies (REMARK) J Natl Cancer Inst 2005 97 16 1180 1184 10.1093/jnci/dji237 16106022

[B30] Pectasides D Papaxoinis G Kotoula V Fountzilas H Korantzis I Koutras A Dimopoulos AM Papakostas P Aravantinos G Varthalitis I Expression of angiogenic markers in the peripheral blood of docetaxel-treated advanced breast cancer patients: A Hellenic Cooperative Oncology Group (HeCOG) study Oncol Rep 2012 27 1 216 224 22025320 10.3892/or.2011.1504

[B31] Livak K Schmittgen T Analysis of relative gene expression data using real-time quantitative PCR and the 2(−DeltaDeltaCt) Method Methods 2001 25 4 402 408 10.1006/meth.2001.1262 11846609

[B32] Van Heugen JC De Graeve J Zorza G Puozzo C New sensitive liquid chromatography method coupled with tandem mass spectrometric detection for the clinical analysis of vinorelbine and its metabolites in blood, plasma, urine and faeces J Chromatogr A 2001 926 1 11 20 10.1016/S0021-9673(01)00993-1 11554404

[B33] Cohen J Statistical Power Analysis for the Behavioral Sciences 1977 New York, NY: Academic Press

[B34] Thall PF Cook JD Dose-finding based on efficacy-toxicity trade-offs Biometrics 2004 60 3 684 693 10.1111/j.0006-341X.2004.00218.x 15339291

[B35] Verheul HM Pinedo HM Possible molecular mechanisms involved in the toxicity of angiogenesis inhibition Nat Rev Cancer 2007 7 6 475 485 10.1038/nrc2152 17522716

[B36] Eskens FA Verweij J The clinical toxicity profile of vascular endothelial growth factor (VEGF) and vascular endothelial growth factor receptor (VEGFR) targeting angiogenesis inhibitors; a review Eur J Cancer 2006 42 18 3127 3139 10.1016/j.ejca.2006.09.015 17098419

[B37] Folkman J Angiogenesis: an organizing principle for drug discovery? Nat Rev Drug Discov 2007 6 4 273 286 10.1038/nrd2115 17396134

[B38] Pappas P Biziota I Marselos M Brasoulis E Evaluation of antiproliferative and molecular effects of vinorelbine and its active metabolite 4-O-deacetyl-vinorelbine on human endothelial cells in an in vitro simulation model of metronomic chemotherapy Eur J Cancer 2008 6 Supplement 9s 138 139 Abstract 533

[B39] Hamaguchi I Huang XL Takakura N Tada J Yamaguchi Y Kodama H Suda T In vitro hematopoietic and endothelial cell development from cells expressing TEK receptor in murine aorta-gonad-mesonephros region Blood 1999 93 5 1549 1556 10029583

[B40] Dales JP Garcia S Bonnier P Duffaud F Meunier-Carpentier S Andrac-Meyer L Lavaut MN Allasia C Charpin C Tie2/Tek expression in breast carcinoma: correlations of immunohistochemical assays and long-term follow-up in a series of 909 patients Int J Oncol 2003 22 2 391 397 12527939

[B41] Dumont DJ Yamaguchi TP Conlon RA Rossant J Breitman ML tek, a novel tyrosine kinase gene located on mouse chromosome 4, is expressed in endothelial cells and their presumptive precursors Oncogene 1992 7 8 1471 1480 1630810

[B42] Laquente B Vinals F Germa JR Metronomic chemotherapy: an antiangiogenic scheduling Clin Transl Oncol 2007 9 2 93 98 10.1007/s12094-007-0018-3 17329220

[B43] Gasparini G Metronomic scheduling: the future of chemotherapy? Lancet Oncol 2001 2 12 733 740 10.1016/S1470-2045(01)00587-3 11902515

